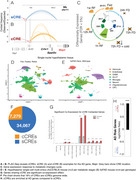# Characterization of Coordinator Cis‐Regulatory Elements ‐ cCREs in the 5xFAD Mouse hypothalamus: Key regulators in Alzheimer's Disease

**DOI:** 10.1002/alz70855_098093

**Published:** 2025-12-23

**Authors:** Josue D Gonzalez Murcia, A. Cristina Rodriguez, Elliot Ferriss, Pablo J Maldonado‐Catala, Christopher Gregg

**Affiliations:** ^1^ University of Utah, Salt Lake City, UT, USA

## Abstract

**Background:**

Alzheimer's disease (AD) is the 5th leading cause of death in adults over 65. Studies show that 40% of AD risk is modifiable, with metabolic factors playing a significant role, but the mechanisms are poorly defined. Metabolic pathways influence chromatin at cis‐regulatory elements (CREs) that control gene expression, suggesting a potential link between metabolism and AD progression. We identified a novel class of CREs, termed coordinator cis‐regulatory elements (cCREs), which form simultaneous regulatory contacts with multiple neighboring genes (Figure 1B), in contrast to canonical singleton CREs (sCREs) that regulate only a single gene (Figure 1A). This study explores the role of cCREs in linking metabolism and AD in hypothalamic tissue.

**Method:**

We employed PLAC‐Seq targeting H3K27ac+ chromatin sites to uncover 3D chromatin structures and active cis‐regulatory contacts across metabolic states (fed, fasted, refed) in hypothalamic tissue of C57BL/6 mice (Figure 1C). To investigate the role of cCREs in AD, we utilized bulk RNA sequencing (*n* = 8 per condition) and single‐nuclei multi‐omics (GEX and ATAC) in both C57BL/6 and 5xFAD mouse models (*n* = 4 per condition) (Figure 1D‐E).

**Result:**

We identified that 18% of CREs in the mouse hypothalamus are cCREs, while 82% are sCREs (Figure 1F). cCREs were associated with neighboring gene co‐expression in response to metabolic changes through two models: (i) a 'mutually exclusive' (ME) model, where only one gene engages the cCRE per cell, or (ii) a 'co‐expression' (CoE) model, where multiple genes are simultaneously regulated by the cCRE (Figure 1G). Importantly, AD risk genes were significantly enriched for promoter contacts with cCREs compared to other genes in the mouse genome (Figure 1H), highlighting cCREs as potential mediators between metabolic pathways and AD pathology.

**Conclusion:**

Our findings reveal a novel gene regulatory mechanism involving cCREs in the mouse hypothalamus that connects metabolism to AD risk. This research provides crucial insights into the impact of metabolic factors on AD progression and resilience, uncovering potential therapeutic targets at cCREs within AD risk loci.